# Genetic Resources of *Olea europaea* L. in the Garda Trentino Olive Groves Revealed by Ancient Trees Genotyping and Parentage Analysis of Drupe Embryos

**DOI:** 10.3390/genes11101171

**Published:** 2020-10-06

**Authors:** Paula Moreno-Sanz, Luca Lombardo, Silvia Lorenzi, Franco Michelotti, Maria Stella Grando

**Affiliations:** 1 Center Agriculture Food and Environment (C3A), University of Trento, Via E. Mach 1, 38010 San Michele all’Adige (TN), Italy; lombluca@yahoo.it (L.L.); stella.grando@unitn.it (M.S.G.); 2 Research and Innovation Centre, Fondazione Edmund Mach, Via E. Mach 1, 38010 San Michele all’Adige (TN), Italy; silvia.lorenzi@fmach.it; 3 Technology Transfer Centre, Fondazione Edmund Mach, Via E. Mach 1, 38010 San Michele all’Adige (TN), Italy; franco.michelotti@fmach.it

**Keywords:** ‘Casaliva’, ‘Frantoio’, *Olea europaea*, parentage analysis, Garda Trentino, genetic diversity

## Abstract

The area of the Garda Lake within the Trentino province (north of Italy) is the northernmost part of Europe where the Mediterranean species *Olea europaea* L. is traditionally cultivated. ‘Casaliva’ is claimed as the main variety traditionally grown in the Garda Trentino area (GT) from which a world renowned niche extra virgin olive oil is produced. Since a dominant presence of ‘Casaliva’ would link the fruit set success and yield to a self-pollination compatibility system, a deep genetic survey of the olive tree population in the GT has been performed with the aim of establishing the actual varietal composition and of understanding from which pollen donor the ‘Casaliva’ olives originate. Forty-four different genetic profiles were observed among the 205 leaf samples collected from 106 ancient trees through the analysis of 20 nuclear microsatellite markers. The varietal composition in modern orchards was also explored and the vast majority of the additional 151 trees analyzed showed the same genotype as the ancient accessions of ‘Casaliva’. The results support the long historical link of ‘Casaliva’ with the GT and, besides a high varietal homogeneity, they also revealed the presence of olive genetic resources essential to fruit production. In fact, the parentage analysis of 550 embryos from drupes of ‘Casaliva’ evidenced that a cross-fertilization system is favored and a list of candidate cultivars most suitable as local pollinizers of ‘Casaliva’ was identified.

## 1. Introduction

**Figure 1:**
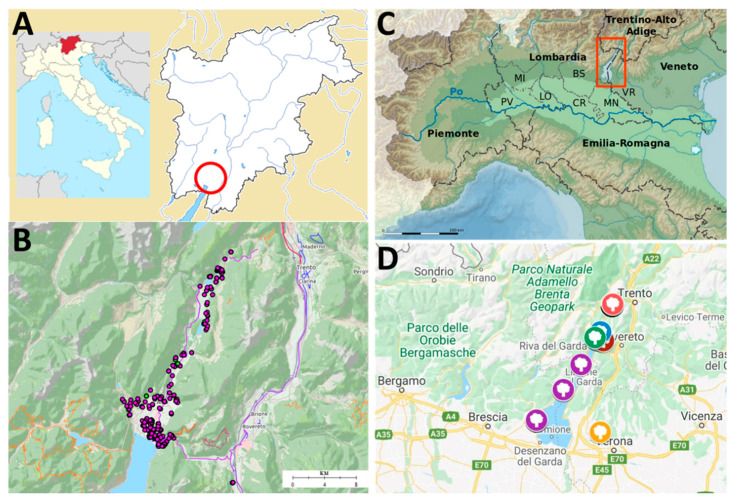
(**A**) Localization of the Trentino-Alto Adige region in Italy, which comprises two provinces: Trentino (to the south) and Alto Adige (to the north). The Trentino area surrounding the Garda Lake where the survey was conducted is evidenced with a red circle (the olive Garda Trentino area or territory). (**B**) Detailed surveyed area showing the localization of the studied olive trees along the banks of the Garda Lake and the Sarca Valley. (**C**) The Garda Lake and its shoreline are divided between the Italian regions of Veneto (to the south east), Lombardy (south-west), and Trentino-Alto Adige (north). (**D**) Local collections from which reference material from the Garda Lake area was collected. Blue: Ischia private collection (22 accessions collected), brown: Olive Research Centre (five accessions collected), green: Chiarani private collection (5 accessions collected), pink: Santa Massenza collection (45 accessions collected), purple: Associazione Interprovinciale Produttori Olivicoli Lombardi (Interprovincial Association of Lombard Olive Producers, seven accessions collected), yellow: Istituto Sperimentale di Frutticoltura of Verona (Experimental Institute of Fruit Growing of Verona), which hosts olive cultivars from the Garda Lake area located in the Veneto region (24 accessions collected). Source of the maps: google maps and Wikipedia.

The area of the Garda Lake within the Trentino province (north of Italy), due to its particular insubric mesoclimate [1,2], is the northernmost part of Europe (beyond the 45th parallel) where the Mediterranean species *Olea europaea* L. is traditionally cultivated. This isolated area is not limited only to the banks of the Garda Lake, but it goes far beyond going up into the Sarca Valley to the lakelets of Toblino and Santa Massenza (Figure 1). 

Despite the climatic events in time, the ancient presence of olive-trees in the Garda Trentino area (GT) has been reported by a number of historical documents (reviewed and collected in [3,4]), where references to the nomenclature of cultivars and their identity were also described. Cultivars such as ‘Casaliva’ (also known as ‘Drizzar’, ‘Casalì’, or ‘Nostran’), ‘Razza’ (also known as ‘Razzo’), ‘Favarol’ (described as similar to ‘Mignolo Toscano’), ‘Dropp’ (also denominated ‘Tombolot’, ‘Fort’, and described as similar to ‘Leccino’), ‘Gargnan’ (‘Gargnà’ or ‘Grignan’, described as similar to ‘Moraiolo’) have been cited in the beginning of the 19th century [3]. ‘Casaliva’ and ‘Razza’ were already then considered cultivars belonging to the ‘Frantoio’ landrace. In fact, recent studies based on genetic fingerprinting through molecular markers have evidenced that these two cultivars are synonyms of ‘Frantoio’ [5,6,7,8].

The study of minor local varieties widespread in small and/or isolated areas with an antique olive growing tradition, such as the GT, becomes of great importance, since this germplasm has likely been carefully selected for a better adaptation to the local environment along the past and may contain traits that could help to cope with hardships and challenges of modern olive growing in a climate change context. Actually, the Italian olive germplasm, estimated to include about 800 cultivars (most of them landraces vegetatively propagated at a farm level since ancient times), is probably underestimated because of the scarce information on less-common minor local varieties [9,10]. The olive germplasm around the Venetian and Lombard Garda Lake shores has been previously described and characterized [5,8,11,12], while no exhaustive characterization of the olive genetic resources in the isolated GT is available.

Nowadays, ‘Casaliva’ is claimed as the main variety traditionally grown in the GT from which a world renowned niche extra virgin olive oil (EVOO) is produced (http://www.wboo.org/worlds-best-olive-oils.html, [2]). In addition to the economic value of the EVOO, olive cultivation in the GT has a synergistic impact in the tourism sector, since the olive groves are mainly made up of ancient and secular trees arranged along spectacular landscape terraces. Therefore, the preservation of a highly important cultural landscape [13] in the GT, in which the olive groves play an outstanding social and economic role, underscores the importance of the environmental preservation of this territory as a model of a High Nature Value farming system (HNV; [14,15,16,17]).

Numerous studies on the surveying, localization and characterization of varieties are being carried out worldwide with the aim of cataloging and preserving olive genetic resources in germplasm banks [8,10,17,18,19,20,21,22]. A pool of genes of agronomical potential and conferring different organoleptic features to the olive oil will thus be available for olive genetic improvement programs [23,24,25]. Precise cultivar identification (traditionally performed through description of morphological, agronomical, elaiographic, biochemical traits and, more recently, through molecular markers) is required to conserve and maintain genetic resources [18].

DNA genotyping by microsatellite markers or simple sequence repeats (SSRs) is the most suitable method for olive genetic variability studies [18,26,27,28]. A different set of markers, tested in true-to-type reference material, have been proposed as standard lists to be used for olive cultivar identification [29,30,31,32], being those within the consensus list of 11 loci proposed by Baldoni et al. [30] for olive genotyping the most used [6,7,8,13,21,28,33,34]. Moreover, the molecular and morphological characterization of the two Worldwide Olive Germplasm Banks (WOGB) of Córdoba [31] and Morocco [32,33] provided an important tool for scientists, nurseries and breeders.

Olive (*Olea europaea* L.) is an anemophilous pollinated species that usually blooms copiously. Nevertheless, the profitability of olive growing is limited by some physiological characteristics related to flower biology, which greatly influence the production potential of the species. Specifically, the conspicuous presence of staminate flowers (with ovaries only partially developed, or absent) on olive trees has been viewed as a mechanism for conserving available resources without affecting the total number of flowers or, thereby, pollen production [35,36]. Additionally, olive cultivars display various levels of self-incompatibility (SI), being somewhere between self-fertility and complete self-sterility. Considering that a certain degree of inter-incompatibility between cultivars has also been described, the identification and use of the most suitable pollinizers is a key point in the strategies of modern olive growing. The choice of pollinizers is also important for varieties considered self-fertile, since experiments carried out on these varieties report higher rates of fruit setting following cross-pollination [35,37,38,39,40]. Moreover, conflicting indications about pollen compatibility in some cultivars are reported as well as contradictory results that have been obtained in different areas and years [41,42,43]. The evaluation of self-incompatibility is therefore crucial from an agronomic and physiological point of view. In this regard, a dominant presence of ‘Casaliva’ in the GT would link the fruit set success and yield to a self-pollination compatibility system.

A deep genetic survey of the olive tree population in the GT has, therefore, been performed with the aim of (i) establishing the current varietal composition of the olive genetic resources of this isolated area and (ii) understanding, through parentage analysis of embryos, from which pollen donors the fruits of ‘Casaliva’ originate.

## 2. Materials and Methods

### 2.1. Experimental Estates and Plant Material

The research was conducted in several olive groves located in the GT (in the Trentino-Alto Adige region, Italy; Figure 1).

Study of the varietal composition: The GT olive local biodiversity was investigated via SSRs. Leaves were collected from 106 ancient olive-trees. An approximate estimation of the range of the age of the investigated trees was calculated by using the algorithms developed by Pannelli et al. [44] (y = 5.2983x + 54.431, where y = years and x = radius at a height of 1.0 m in cm) and Arnan et al. [45] (y = 2.1125x + 88.925, where y = years and x = diameter) (both references in [17]). The trunk circumference at 1 m above the ground (perimeter breast height: PBH) was only measured in the trees with the lowest and highest trunk circumference. The radius and diameter were calculated from the circumference formula: C = 2πr or C = dπ (where C = circumference (PBH), r = radius and d = diameter). Samples from different parts of a tree (branches, suckers, basal sprouts) were collected in the case of the oldest trees and, in a few cases, when morphological differences were visually noticed between the canopy and the suckers (to verify if it was due to a juvenile state or to a real graft) or in the canopy within the tree, reaching a total of 205 samples (Table S1). In modern orchards (30 years old approximately) leaves were collected from the canopy of 151 plants, this time only one sample per tree was collected. The survey was very selective with the aim of capturing the highest possible diversity. In this regard, plants were chosen according to morphological and bio-agronomic characters, such as leaf and drupe morphology, the attitude of the shoots and plant size, which made them peculiar when compared to ‘Casaliva’-type trees.

A total of 108 additional accessions from local germplasm collections, which host mainly accessions from the Garda Lake olive growing area, were genotyped for varietal identification purposes (Figure 1D, Table S2). Furthermore, thirteen true-to-type accessions from the Worldwide Olive Germplasm Bank of Córdoba, Spain (hereafter WOGBC) [31] and three from the Italian germplasm collection of the Consiglio per la Ricerca in agricoltura e l’analisi dell’Economia Agraria-Centro di Ricerca per l’Olivicoltura, Frutticoltura, Agrumicoltura of Rende, Italy (hereafter CREA-OFA) [6] were included in order to perform a better data harmonization for comparison with these databases (Table S3). The public joint database of the WOGB of Marrakech (Morrocco) and Córdoba (Spain), with 14 SSR markers analyzed in common with our study, was also consulted [32], as well as the Italian database from Perugia University (hereafter UNIPG) [7], with 9 SSR markers in common.

Parentage analysis: Embryos from a total of 550 drupes of ‘Casaliva’ were isolated to perform a parentage analysis. A total of 153 drupes were randomly collected from plants throughout the GT, in the localities of Monte Brione, Arco and from an olive mill, while 397 drupes were collected from 12 molecularly characterized trees of ‘Casaliva’ located in five different localities: Fraveggio (2 plants, modern olive grove), Linfano (2 plants, modern olive grove), Monte Brione (4 plants, mature olive grove), Arco (2 plants, mature olive grove) and Torbole (2 isolated plants not intended for commercial purposes). Meanwhile, an indirect assessment of the self-fertility of ‘Casaliva’ was realized by bagging a couple of twigs in blossom, bearing a hundred inflorescences each, from six plants. Self-pollination in olives formed inside the bags was eventually ascertained through molecular markers and parentage analysis.

### 2.2. Genotyping of Trees and Drupes via SSR Analysis

Fresh young leaves and early harvested drupes (collected in September) were collected in the field and preserved at −80 °C until DNA analysis. Sampled leaves were dried in liquid nitrogen and grounded to a fine powder for DNA extraction. For each drupe the mesocarp was manually removed and the endocarp was carefully opened by using a wheel pipe cutter of 20 mm capacity in order to take out the internal seed. Successively, the tegument of the seed was removed, the endosperm carefully opened with a scalpel and finally the embryo was isolated. Isolated embryos were lyophilized for 12–24 hours for DNA extraction. Total genomic DNA was extracted using a commercial kit (plant and fungi DNA isolation kit, NORGEN Biotek Corp., Canada). The DNA quality was checked with a NanoDrop 8000 spectrophotometer (Thermo Scientific, Waltham, MA, USA). 

Analysis of the varietal composition: A set of 21 labeled (6-FAM, HEX or NED) microsatellite markers (SSRs) were used for genotyping of local germplasm collections, reference material and ancient olive populations: DCA03, DCA04, DCA05, DCA10, DCA11, DCA14, DCA16, DCA18 [46]; UDO03, UDO05, UDO12, UDO28, UDO39, UDO43 [5]; GAPU59, GAPU89, GAPU71A, GAPU71B, GAPU101, GAPU103A [47] and EMO90 [48]. 

Parentage analysis: A subset of 11 markers (DCA03, DCA10, DCA16, DCA18, GAPU71B, GAPU89, GAPU101, GAPU103A, UDO05, UDO39, and UDO43), chosen because of their higher polymorphism and heterozygosity according to the values obtained for the set of genotypes explored as possible parents (see point 2.6), were used for the analysis of the 550 embryos from drupes of ‘Casaliva’ for parentage analysis. 

Polymerase chain reactions (PCRs) were carried out in 15 μL final volume using a thermal cycler (GeneAmp PCR System 9700 Applied Biosystems Inc., Foster City, CA, USA). The reaction mixture was composed of 10 ng of template DNA, 1X PCR buffer, 2 mM MgCl_2_, 100 µM of each dNTP, 0.2 μM of forward and reverse primers, and 0.5 U AmpliTaq GoldTM DNA Polymerase (Applied Biosystems Inc, Foster City, CA, USA). The PCR thermal profile was programmed as follows: a first step at 95 °C for 5 min, 35 cycles at 95 °C for 20 s, 55 °C for 30 s, and 72 °C for 30 s (45 sec for UDO05). The last step included 10 min of incubation at 72 °C. The success of the amplifications was checked in 1% agarose gels. PCR products (0.5 µl) were mixed with 9.3 µl of formamide and 0.2 µl of the GeneScanTM 500 ROX^®^ Size Standard (Life Technologies, Carlsbad, CA, USA). The DNA fragments were denatured and separated on an ABI PRISM Genetic Analyzer 3130xl (Applied Biosystems Inc., Foster City, CA, USA). The allelic assignment was performed using GeneMapper v.4. (Applied Biosystems™, Foster City, CA, USA) Standardization of raw data was conducted in comparison to the authenticated molecular profiles of true-to-type accessions from the WOGBC.

### 2.3. Genetic Diversity Analysis

The number of alleles (Na), number of effective alleles (Ne), allele frequencies, expected (H_e_) and observed (H_o_) heterozygosity, polymorphic information content (PIC) and probability of identity (PI) were calculated using GenAlEx v.6.5 (https://biology-assets.anu.edu.au/GenAlEx/Welcome.html) [49,50]. The probability of null alleles (*r*) was calculated using Cervus 3.0.7 (http://www.fieldgenetics.com/pages/home.jsp) [51,52].

### 2.4. Cluster Analysis

Cluster analysis was performed from a presence-absence matrix constructed using the allele values obtained for all SSR genotypes of the ancient populations’ set and of the local, national and international reference material analyzed. The value ‘1’ was assigned to the presence of a certain allele, and ‘0’ to its absence. When amplification failed for a certain locus in a genotype, the integer code ‘9’ was set for all alleles of that locus as missing data. Genotype 61 was removed from the analysis due to a percentage of missing data higher than 50% (Table S4). The cluster analysis was performed using NTSYS-PC v.2.2 software (http://www.appliedbiostat.com/ntsyspc/ntsyspc.html) [53,54] for 19 out of the 21 SSR markers analyzed (UDO03 and UDO28 were excluded because they presented more than 40% of missing data). The SimQual module of this software was used to perform a cluster analysis based on the Unweighted Pair-Group Method with Arithmetic Averages (UPGMA) algorithm using Dice’s similarity coefficient. The correlation coefficient between the similarity matrix and the cophenetic values matrix was computed (Coph and MxComp modules of the software) to test the goodness-of-fit for the cluster analysis. 

### 2.5. Analysis of the Population Structure

A Bayesian clustering algorithm implemented with STRUCTURE 2.3.4 [55] was performed to sort individuals into K clusters (subpopulations) according to their genetic similarity. The analysis was performed with the dataset of 43 unique profiles obtained for the ancient populations’ set of samples (genotype 109 was excluded for being equal to genotype 70, but with an extra allele at locus UDO03) and the 672 genotypes from WOGB databases once harmonized [32] for the markers analyzed in common. Germplasm from the local collections was not included. An admixed model with allele frequencies correlated was assumed and no prior population information was set up. Ten independent runs for K values ranging from 1 to 10 were performed with a burn-in period of 50,000 and a number of MCMC replicates after burn-in of 500,000. STRUCTURE output was further analyzed with Structure Harvester [56] to estimate the best K by the ΔK method [57]. Then CLUMPP [58] was used to permute clusters output by the 10 independent runs of STRUCTURE for the selected K value, so that the clusters align across runs, and to get a final Q-matrix. DISTRUCT [59] was used for graphical representation of the aligned cluster assignments for a single K value. A threshold of the coefficient of membership of Q > 80% was considered to unequivocally assign a genotype to a group. Individuals with intermediate admixture coefficients (Q < 80%) were deemed ‘admixed’. CLUMPAK server [60] was used for graphical representation of the aligned cluster assignments for different K values.

### 2.6. Parentage Analysis

The computer software package Cervus 3.0.7 [51,52] was used for this purpose. For each offspring tested, the software calculates the log of the odds or likelihood ratio (LOD) scores for each candidate parent and the most-likely candidate parent is identified with a pre-determined level of confidence, or is left unassigned. The LOD score measures the likelihood that the candidate parent is the true parent divided by the likelihood that the candidate parent is not the true parent. The laboratory typing error is also considered and statistical confidence is determined for assigned paternities through simulation. 

In our experiment, the number of candidate parents (pollinizers) was directly derived from the results of the genetic survey of the local biodiversity of olive trees in the GT. Default values were adopted for the parameters ‘proportion of loci mistyped’ and ‘error rate in likelihood calculations’. The relaxed and strict confidence levels were set to 95% and 99%, respectively.

### 2.7. Statistical Analysis

Parametric one-way analysis of variance (ANOVA) was performed, after having tested the normality and homoscedasticity of residuals, to assess significant differences in the percentage of self-pollination according to the sampling area and in the percentage of deteriorated embryos per olive tree characteristics. Post-hoc multiple means comparison at a confidence level of 95% and 99% were made using Tukey’s honestly significant difference (HSD) test through PAST software v.2.12 (http://priede.bf.lu.lv/ftp/pub/TIS/datu_analiize/PAST/2.17c/download.html) [61].

## 3. Results

### 3.1. Germplasm Collections 

The 21 SSR markers used for genotyping the reference material from the WOGBC, the CREA-OFA, and the 108 olive accessions from the Garda Lake germplasm collections were selected as those showing the most robust results among an initial set of 24 tested markers, which comprises the standard set of 11 SSRs [30], together with 13 additional markers also used for characterization of the CREA-OFA germplasm collection and/or the WOGBC (Table S3). 

Samples of the 13 true-to-type olive cultivars from the WOGBC analyzed presented similar genetic profiles to those reported by the database of origin for the 17 SSRs analyzed in common; although a high percentage of homozygous genotypes, instead of heterozygous as reported by the WOGBC [31], was observed for locus DCA11, which could be due to the presence of null alleles (*r*) in our analysis (*r* = 0.4454 for DCA11, Tables S5 and S3). The analysis of these accessions in our laboratory allowed an accurate harmonization of the WOGB databases [31,32] with the aim of using them for varietal identification purposes (Table S3).

In the case of the three accessions from the CREA-OFA germplasm collection, the size difference between the minor and the major alleles for some of the nine loci analyzed in common was not the same as that of the database of origin [6]. Anyway, the genetic profiles we obtained of ‘Leccino’ and ‘Frantoio’ from this database were identical to the ones obtained for the true-to-type accessions of these two cultivars from the WOGBC, respectively (‘Frantoio’ of the CREA-OFA was heterozygous for DCA10, instead of homozygous as the accession from WOGBC). For the nine SSRs in common with the CREA-OFA database, ‘Casaliva’ and ‘Frantoio’ presented the same genetic profile, but when the number of loci was extended to 21 a difference was observed for the UDO43 major allele (Table S3). The ‘Leccino’ genotype was excluded for data harmonization of this database due to the incongruent results found with the database of origin; only genotypes of ‘Frantoio’ and ‘Casaliva’ were used.

**Figure 2:**
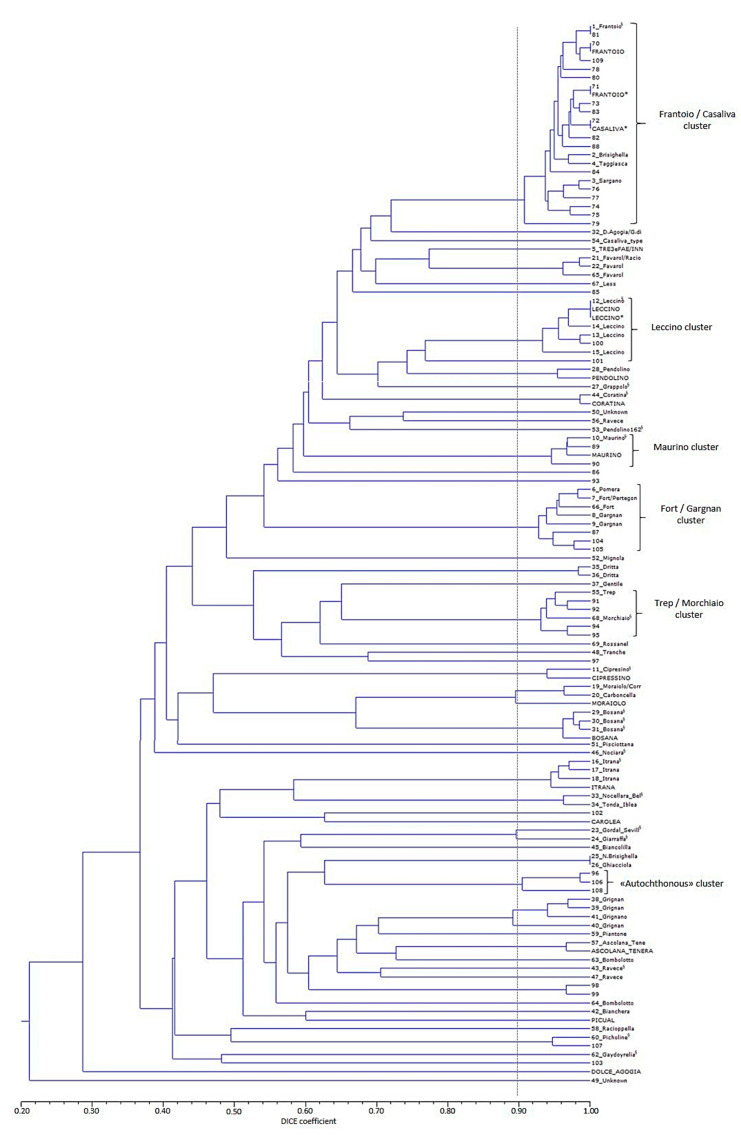
Dendrogram, generated by applying the UPGMA method, using Dice’s coefficient matrix, of the different genotypes identified among the local collections (SSR code according to Table S2 followed by the accession name) and the olive ancient populations (only SSR code). Reference cultivars genotyped from WOGBC (capital letters) and CREA-OFA databases (capital letters with *) are also included. The dotted grey line indicates 90% similarity (threshold arbitrary chosen). The cophenetic correlation coefficient was 0.875. Genotypes of local collections with § superscript show the cultivar name they have been identified with, instead of the accession name.

The genotyping of the 108 accessions collected from the local germplasm collections (Table S2) as a representation of the varietal diversity of the whole Garda Lake olive area revealed 69 different molecular genetic profiles (Table S4). Forty-nine of them were represented by only one accession, while the remaining 20 genotypes were present in more than one accession (Table 1 and Table S2). In some cases, samples with a similar accession name presented different genotypes (this occurs, for example, with accessions named ‘Favarol’, ‘Pertegon’, ‘Peranzana’, among others, see Table S2), although most of them presented a high degree of similarity (Figure 2)

On the other hand, samples with the same SSR genotype, in some cases, presented different accession names (see profiles 1, 7, 19, 21, 25, 27, 28, 32 and 68 in Table S2). After comparison with WOGB from Córdoba and Morocco [31,32], CREA-OFA [6] and UNIPG [7] databases, matches were found for 23 genetic profiles. The germplasm was assigned to the Italian cultivars ‘Ascolana Tenera’, ‘Bosana’, ‘Cipresino’, ‘Frantoio’, ‘Giarraffa’, ‘Itrana’, ‘Leccino’, ‘Nociara’, ‘Nocellara del Belice’, ‘Pendolino’, ‘Grappolo’, ‘Coratina’, ‘Maurino’, ‘Morchiaio’, ‘Moraiolo’ and ‘Ravece’ and also to cultivars originally from Croatia, France, Greece and Spain (‘Plementa Bjelica’, ‘Picholine’, ‘Gaydoyrelia’ and ‘Gordal Sevillana’, respectively) (Table S2, Figure 2), most of the times confirming the given accession name. The three accessions with the genotype matching with the ‘Plementa Bjelica’ one were all collected with the accession name ‘Bianchera’ and, according to the database consulted, ‘Plementa Bjelica’ is a synonym of this cultivar. In the case of ‘Peranzana’, ‘Leccio del Corno’ and ‘Carboncella’, these accession names resulted in being confirmed synonymies of prime cultivar names (‘Bosana’, ‘Grappolo’ and ‘Moraiolo’, respectively). The genetic diversity parameters and statistics for the set of accessions of the local collections are shown in Table S5 and Table 1.

### 3.2. Ancient Populations of the Garda Trentino Area

Forty-four different genetic profiles have been observed among the 205 accessions, which were collected from 106 ancient olive trees located in the Trentino area of the Garda Lake (Tables S1 and S4). Thirty-five of these genotypes were represented by only one tree, while the remaining 9 genetic profiles were present in more than one tree (Table 1 and Table S1). All SSR markers were polymorphic, the number of alleles per locus ranged from 2 (UDO03) to 12 (UDO43 and GAPU103A) with 131 total alleles (in line with the total number of alleles reported for other databases from Italy, Table 1) and a mean number of alleles per locus of 6.5. According to their polymorphic information content (PIC), the most informative locus was GAPU103A (PIC = 0.769) and the least was UDO03 (PIC = 0.154). In fact, these two markers presented the lowest and highest probability of identity (PI), respectively (Table S5). The set of markers used for cultivar identification in the GT area showed a high discrimination power according to the cumulative PI value (4.4 × 10^−16^), which was coherent with that obtained for the set of accessions from local germplasm collections genotyped and lower than that obtained for the characterization of 561 accessions with 12 SSR markers (eight in common with this study) of the WOGBM collection (Table 1). The mean PIC value was a bit lower compared to the range of values obtained in different databases (Table 1), due to the presence of several molecular genetic variants of ‘Frantoio‘ and ‘Frantoio’-related cultivars (Tables S1 and S4, Figure 2), as we will show below. In fact, when these variants are excluded from the genetic analysis the mean PIC value reaches a value comparable to that of the other databases (0.619). The H_e_ ranged from 0.171 (UDO03) to 0.806 (GAPU103A) with a mean value of 0.638. The average H_o_ was slightly higher (0.687) and ranged from 0.168 (UDO03) to 1.00 (GAPU101). Despite that the H_e_ of the ancient populations’ set was a bit lower than that from the accessions of local germplasm collections (which represents the diversity of the olive cultivars present in the whole Garda Lake area), the H_o_ in ancient populations was higher and was also congruent with that observed for the Italian and WOGBC databases (Table 1). The most informative loci were DCA03, DCA16, DCA18, GAPU101, GAPU103A, GAPU71B, UDO43 and EMO90, according to the highest H_o_ and PIC and the lowest PI values for each marker. The estimation of the presence of null alleles was over 25% for markers DCA10, DCA11, UDO03 and UDO39 (Table S5). All genetic diversity parameters calculated are shown in Table S5 and Table 1.

Only four genetic profiles out of 44 matched with those found in the local collections: profiles 5, 11 (a molecular variant of ‘Cipressino’), 12 (‘Leccino’ true-to-type) and 41, and another eight, (24 if molecular variants are considered,) with the other databases (‘Frantoio’, ‘Casaliva’, ‘Maurino’, ‘Razzaio’, ‘Picholine’, ‘Rosselino’, ‘Chalkidikis’, see Table S1 and S4). Therefore, taking into account all molecular variants, cultivar identification was possible for 63.6% of the genotypes identified among the ancient olive populations. A total of 128 accessions showed the ‘Frantoio’/‘Casaliva’ reference profile(s) (SSR codes 70, 71 and 72 in Table S2, Figure 2), which accounted for 62.4% out of the 205 samples analyzed. Twenty-five additional accessions (12.2% of the total) presented a genetic profile, which varies only for one or two alleles (in some cases a homozygous profile instead of heterozygous, or vice versa, regarding that of the ‘Frantoio’/‘Casaliva’ reference profile for one or two loci) that were considered as ‘Frantoio’/‘Casaliva’ molecular variants. Altogether, 74.6% of the 205 accessions analyzed presented a genetic profile clustering in the ‘Frantoio’/‘Casaliva’ group (Figure 2). The remaining genotypes were represented by a number of accessions that ranged from one to six, that is 0.48–2.9% of the total. Variants with over 90% of similarity with ‘Leccino’, ‘Maurino’, ‘Picholine’ and ‘Morchiaio’ were also found, and with the ‘Fort’/’Gargnan’ cluster and 55-’Trep’ among the genotypes of the local collections (Figure 2). Genotypes 96 (trees with accession names of ‘Favarol’ and ‘Trep’), 106 and 108 (trees collected as putative ‘autochthonous’) also grouped together with a high level of similarity (Figure 2).

At the level of the analyzed trees (*n* = 106), no accession name was available for 27 of them, while the remaining 79 trees were collected under the following accession names: ‘Casaliva’ (38), ‘Razza’ (16), ‘Assurgente type’ (5), ‘Autochthonous’ (5), ‘Leccino’ (3), ‘Favarol’ (2), ‘Frantoio’ (2), ‘Trep type’ (2), ‘Ancient’ (1), ‘dwarf plant’ (1), ‘Olif de Bòtes’ (1), ‘Picholine’ (1), ‘Regina del Garda’ (1) and ‘Regina del Lago’ (1). All ‘Leccino’ and ‘Frantoio’ trees presented the same genotype among them (Table S1, Figure 2; SSR code 12 and 70, which matched with ‘Leccino’ and ‘Frantoio’ true-to-type, respectively). The two trees collected as ‘Favarol’ clustered distantly from the ‘Favarol’ group of accessions from local collections (Figure 2). One of them clustered with the ‘Autochthonous’ group instead, while the other was identified as ‘Chalkidikis’ (Table S1). Thirty-one (81.6%) of the ‘Casaliva’ trees presented the ‘Frantoio’ reference genotype, while the profile of the remaining seven was that of ‘Frantoio’ molecular variants (profiles 74 to 80). Among ‘Razza’ trees, thirteen showed the ‘Frantoio’/‘Casaliva’ genetic profile (81%) and three the genotypes of ‘Frantoio’ molecular variants (Table S1).

Two or three samples from different branches of each tree were collected in 51 out of the 106 analyzed trees to check if grafting events had occurred at the level of the branches. Additional samples from suckers and/or basal sprouts were also collected in 30 other olive trees to check the use of rootstocks (Table S1). Different genotypes were observed between branches of the same tree in two cases: AGT008, named as ‘Assurgente type’, and AGT036, named as ‘Regina del Garda’, but in this last case the only difference between genotypes was a mutation at the UDO43 major allele (Table S1). On the other hand, different genetic profiles were identified between samples from the branches and the suckers/basal sprouts for 15 trees among the 30 for which both parts of the tree were analyzed (Table S1). Five of these 15 trees were collected under the name ‘Autochthonous’ (Table S1) and all of them, except one, presented the profile 106 in the crown of the trees while that of the basal part was attributable to ‘Frantoio’ (profiles 70, 71, 83 and 84). All these trees were located in the Monte Brione area. Trees named as ‘Razza’ or ‘Casaliva’ presented the ‘Frantoio’/‘Casaliva’ genetic profile in the crown and genotypes of ‘Frantoio’ variants, 95 or 105 were identified in the basal part. The ‘Trep type’ tree presented the profile 96 in the aerial part and the ‘Frantoio’ one in the basal part. ‘Unknown’ trees, except one, were grafted into ‘Frantoio’ or ‘Frantoio’ variants. It is noteworthy that among the five trees with ‘Frantoio’ genotype in the canopy two were putatively grafted into a molecular variant of ‘Frantoio’ and one tree (GT068) with the genotype of the reference ‘Casaliva’ in the canopy showing a ‘Frantoio’ molecular variant profile at the base.

**Figure 3:**
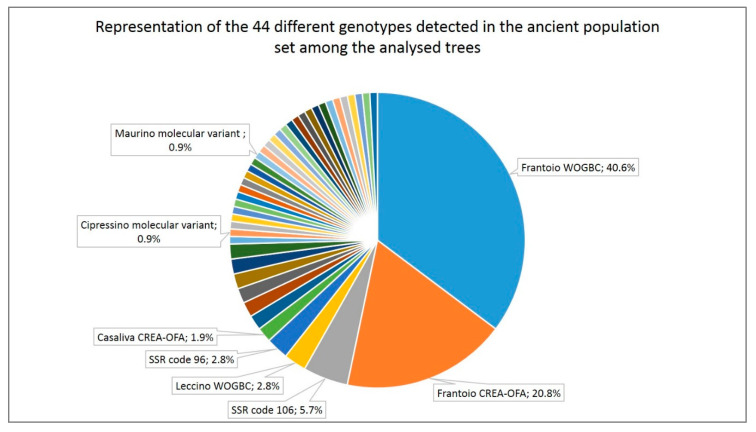
Representation of the 44 different genotypes identified in the ancient populations’ set of samples among the 106 trees analyzed. Data label only present for the identified genotypes and for those most representative among the unknown ones.

Among the 89 trees with a single genotype, 75.3% showed a genotype of the ‘Frantoio’/‘Casaliva’ group, from them 55.2% were collected as ‘Casaliva’, 21% as ‘Razza’, 17.9% unknown and 6% with other accession names (such as ‘Frantoio’, ‘Olif de Bòtes’, ‘Ancient’). If all trees are included, 61.3% of the 106 analyzed trees presented ‘Frantoio’ genotype (31 named ‘Casaliva’, 12 ‘Razza’, two ‘Frantoio’, two ‘autochthonous’, one ‘Ancient’, one ‘Olif de Bòtes’, one ‘Trep type’ and 15 with an unavailable or unknown name), which reach 63.2% when trees with the ‘Casaliva’ genotype, lying in the same cluster as ‘Frantoio’ (Figure 2), were included. Each one of the remaining genotypes was present in a range of 0.9% (most of them) to 5.7% of the trees (Figure 3). 

Each ‘Frantoio’/’Casaliva’ molecular variant was detected mainly in only one tree (maximum two) and they were collected as ‘Casaliva’, ‘Razza’, ‘Autochthonous’ or no accession name was available. 

According to the foregoing, ‘Casaliva’ and ‘Razza’ cultivars are synonyms of ‘Frantoio’, an old genotype, which shows a wide intra-varietal variability at SSR loci. Nevertheless, despite that in the present study a systematical morphological and agronomical description of the trees has not been performed, some phenotypic differences were appreciated between ‘Razza’ and ‘Casaliva’ trees. ‘Razza’ trees are larger than ‘Casaliva’ ones with smaller olives, with a more alternating productivity, longer branches with a less thick foliage and a more upright appearance. However, one of the ancient trees, collected as ‘Razza del Lenzimot’ (AGT012), presented intermediate characteristics between ‘Razza’ and ‘Casaliva’.

### 3.3. Age Estimate of the Ancient Trees

The age estimation obtained was similar with both methods used. According to the lowest (1.78 m) and highest (6.28 m) PBH measures, the age of the analyzed trees ranged from 208 to 511 years according to [45] and from 204 to 584 years depending on [44], confirming the secular character of the investigated trees. The trunk diameter ranged from 0.57 to 2.0 m.

### 3.4. Modern Orchards

A subset of seven SSR markers (DCA03, DCA16, DCA18, EMO90, GAPU71B, GAPU103A, UDO43) was chosen for screening samples from 151 young trees (around 30 years old) with the aim of determining if the varietal composition on modern orchards (in terms of the presence of a predominant cultivar and representation of other minor ones) was similar or different to that observed among the ancient olive populations present in the GT. This subset of markers was chosen according to the loci (analyzed in the ancient populations’ set) that presented the highest PIC values, low null allele probability (Table S5) and/or low amplification failure. Fifty different genetic profiles were identified with this set of markers (Table S6), 39 represented just by one tree and 11 by more than one. Comparison of the obtained genotypes with the local and ancient populations SSR profiles, the reference material and with the consulted databases allowed cultivar identification of 20 of them (40%; Table S6). A total of 46% of the analyzed trees presented the same genotype, which matched with that of ‘Frantoio’, and another 28% of them showed a genetic profile of ‘Frantoio’ molecular variants, adding all this 74% of the trees (Figure S1). 

Taking into account that the survey of trees was selective to get the maximum diversity, the real representation of ‘Frantoio’ genotype in the modern orchards is likely higher, being the predominant cultivar. The remaining genotypes were only represented in a range between 0.7–5.3% of the analyzed trees. 

### 3.5. Analysis of the Population Structure

**Figure 4:**
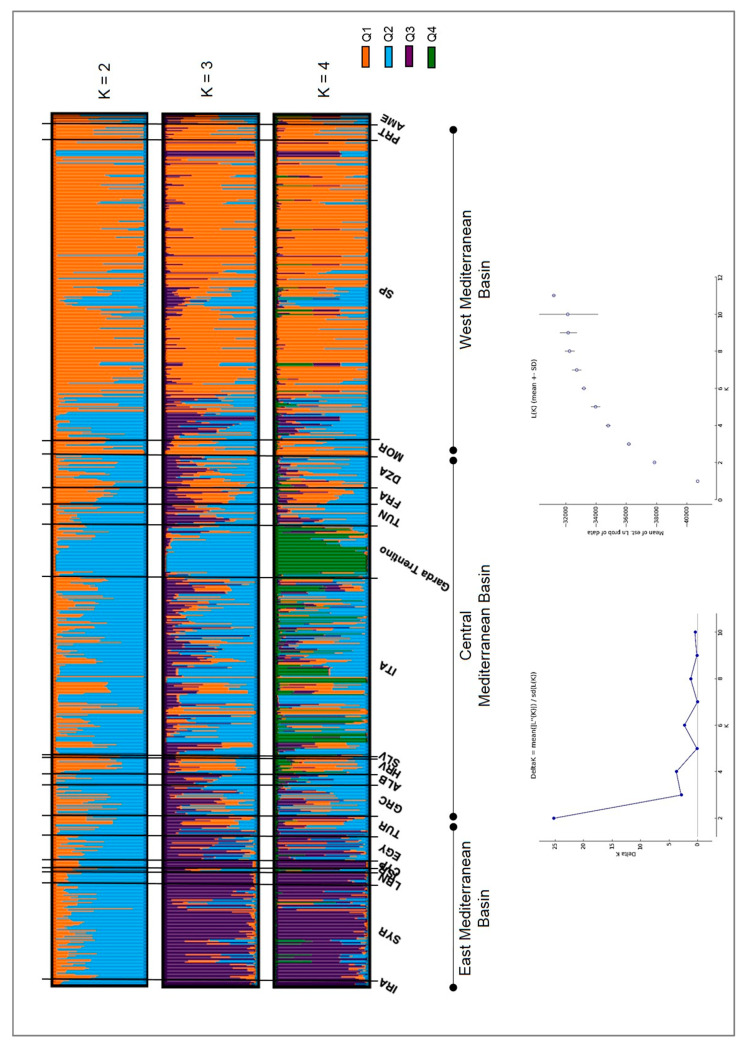
Bayesian inference of population structure (based on 14 nuclear microsatellite markers: DCA 03, 04, 05, 10, 11, 16 and 18; EMO90; GAPU 59, 71A, 71B, 101 and 103; UDO43) in the Mediterranean Basin including genotypes from the joint database of the WOGB of Córdoba and Morocco (*n* = 672; [32]) and the genotypes of the Garda Trentino area (*n* = 43) based on K = 2 to K = 4 subdivisions. The geographical origin of the samples is specified according to [32]: IRA: Iran, SYR: Syria, LBN: Lebanon, ISR: Israel, CYP: Cyprus, AGY: Egypt, TUR: Turkey, GRC: Greece, ALB: Albania, HRV: Croatia, SLV: Slovenia, ITA: Italia, TUN: Tunisia, FRA: France, DZA: Algeria, MOR: Morocco, SP: Spain, PRT: Portugal, AME: America.

The most pertinent level of population subdivision according to Evanno’s ΔKs statistics was K = 2. Minor signals of population stratification were also detected for K = 4, 6 and 8 (Figure 4). 

The two subpopulations at K = 2 mainly distinguished genotypes putatively from the Western Mediterranean Basin (Q1, mostly constituted by the Iberian Peninsula and Morocco genotypes; Figure 4, Tables S7 and S8) from putative genotypes from the Eastern and Central Mediterranean Basin (Italy, Syria, Lebanon, Greece, Algeria, Iran, Turkey and some from Spain, such as Cirujal, Arbequina, Blanqueta, among others; Q2 in Figure 4, Tables S7 and S8). The admixed group was made up of 40% of the genotypes analyzed. All putative French genotypes, except one, were admixed. A high percentage of admixture was also observed for cultivars putatively from Algeria, Egypt, Greece, Croatia, Italy, Morocco and Syria (Tables S7 and S8). Thirty-two out of the 43 GT genotypes clustered within Q2 (among them those within the ‘Frantoio’/‘Casaliva’ group, Figure 2), remaining in the admixed group genotypes identified as ‘Leccino’ (SSR code 12, 100), ‘Picholine’ (SSR code 107), ‘Chalkidikis’ (SSR code 102), the ‘autochthonous’ group (SSR codes 96, 106, 108, see Figure 2) as well as others that were collected without any accession name or as ‘Favarol’ or ‘Trep’ (Tables S1, S7 and S8). Six out of the eleven admixed genotypes were more represented by the Q2 ancestor population, with a membership coefficient ranging from 0.60 < Q < 0.80.

At K = 3 the Western Mediterranean Basin group (Q1) remained stable, while differentiation of genotypes putatively from the Central and Eastern Mediterranean basin occurred, splitting into Q2 and Q3, respectively. Q3 (Eastern Mediterranean) was mainly constituted by cultivars from Syria, Lebanon, Iran, Egypt and Cyprus, as well as two Italian and a few Spanish ones, while Q2 (Central Mediterranean Basin) was mostly made up of Italian cultivars; in fact 33 out of the 43 genetic profiles of the Garda Trentino laid in this group (the 16 genotypes within the ‘Frantoio’/‘Casaliva’ group (Figure 2) and another 17, such as those identified as ‘Leccino’, ‘Razzaio’, ‘Rosselino’ and ‘Maurino’ among others). The remaining 10 genotypes were admixed (among them those identified as ‘Cipressino’, ‘Picholine’, ‘Chalkidikis’ or the ‘autochthonous’ group in Figure 2). The admixture cluster slightly increased at K=3 (46% of the total genotypes) especially due to the shift of putative genotypes from the Central Mediterranean Basin (Figure 4, Tables S7 and S8). All cultivars putatively from France were admixed, as well as a high percentage of genotypes from Algeria, Greece, Croatia, Italy, Morocco, Tunisia and Turkey.

At K = 4 the Western (Q1) and Eastern (Q3) Mediterranean Basin groups remained stable, while part of the Italian cultivars constituting the Central Mediterranean (Q2) group differentiate into a separate subpopulation (Q4), which was constituted by 26 out of the 43 genotypes of the GT area (‘Frantoio’/‘Casaliva’ group, Figure 2, and ‘Leccino’, among others, Table S7 and S8) together with other Italian true-to-type cultivars from Tuscany such as ‘Frantoio’, ‘Pendolino’, ‘Leccino’, ‘Maurino’, ‘Mignolo’ and ‘Grappolo’. Only a molecular variant of ‘Cipressino’ (SSR code 11) of the GT area laid in Q3. Italian true-to-type cultivars, such as ‘Cipressino’, ‘Moraiolo’, ‘Bosana’ and ‘Rosciola’, and a few varieties from Spain (i.e., Blanqueta, Arbequina), Albania, Algeria, Egypt, Greece, Morocco, Syria, Tunisia and Turkey constituted the Central Mediterranean Basin cluster (Q2) and among the Italian cultivars different regions were represented. The admixed group remained quite stable (44% of the total genotypes) with all cultivars putatively from France again within this cluster, together with a high percentage of others putatively from Algeria, Croatia, Italia, Morocco, Tunisia and Turkey (Figure 4, Tables S7 and S8).

At K = 5 and K = 6, for one of the groups the highest coefficient membership did not reach the threshold (Q > 0.80), so they were not further explored, nor were the successive levels of population subdivision.

### 3.6. Deteriorated Embryos

The removal of the endocarps has allowed us to ascertain a high presence of seeds with deteriorated embryos (overall 35.29%). The percentage of deteriorated embryos was generally lower, on average 28.36%, in the young olive groves, rising up to 34.88% in the old olive groves and to 49.09% in the isolated trees not intended for commercial production. These differences were statistically significant (Figure S2). 

### 3.7. Parentage Analysis

**Figure 5:**
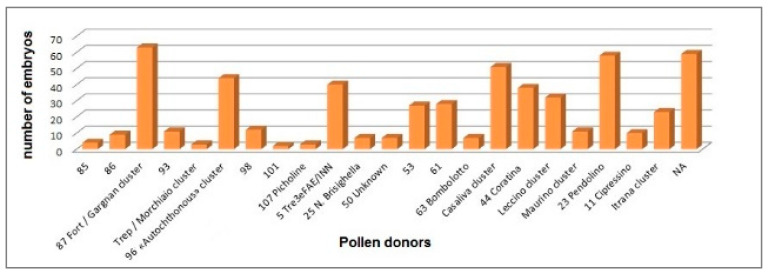
Number of embryos (*n* = 550) from the mother trees of “Casaliva” cv. assigned to putative pollen donors by 11 simple sequence repeats (SSR) markers. NA: not assigned. Genotype codes correspond to SSR codes reported in Table S4.

The results obtained on 550 olive embryos (Figure 5) from mother trees of ‘Casaliva’, showed a low self-fertilization rate in conditions of free pollination for this cultivar (9.45%), despite an almost-monoculture of this variety in the GT proven by this study. However, a great variability in the percentage of self-pollination was recorded according to the sampling area, ranging from 0% in the embryos collected in Linfano, to 6.26% in those from Fraveggio, to 9.11% in those from Arco, to 10.06% in those from Monte Brione, to 22.97% in those from Torbole. In particular, the percentage of embryos from ‘Casaliva’ auto-pollination found in Torbole was statistically higher than those obtained in other areas (Figure S3).

The most effective pollinizers resulted to be profile 87 from the ‘Fort’/‘Gargnan’ cluster (according to the SSR codes in Table S4, Figure 2) and ‘Pendolino’ for 11.45% and 10.54% of the analyzed embryos, respectively, followed by ‘Casaliva’ (9.45%), profile 96 (from the ‘Autochthonous’ cluster, 8%), profile 5 (‘Tre3eFAE/INN’, 7.27%) and ‘Coratina’ (6.90%). Overall, 22 different putative pollen donors have been identified within the 72 tested varieties (Figure 5). Eventually, the percentage of unassigned parents (10.74%) might indicate the presence in the GT or in neighboring cultivation areas of genotypes not captured in our study.

**Figure 6:**
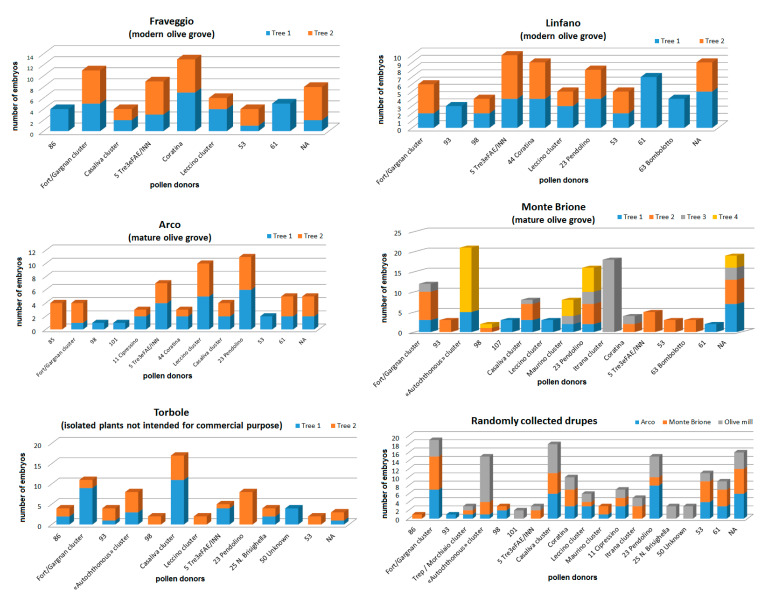
Parentage assignment per sampling area. NA: not assigned. Genotype codes correspond to SSR codes reported in Table S4.

Considering parentage assignment based on the sampling area (Figure 6), some recurring putative pollen donor cultivars such as profile 87, profile 5, profile 53 and ‘Leccino’ were highlighted throughout the GT. On the contrary, some putative fathers like profile 85 and ‘Itrana’ were recorded only on a single plant in a single area.

A single case of polyembryonic seed (two embryos within a unique endosperm) has been detected (Figure S4). The SSR profile revealed that the twin embryos were genetically identical, originated by a ‘Casaliva’ × ‘Cipressino’ cross.

Only eight integral embryos from drupes collected from the paper bags wrapping flower branches of ‘Casaliva’ trees could be molecularly analyzed to verify intra-plant selfing capability. Self-pollination was confirmed in all embryos, supporting a certain degree of self-fertility of this cultivar.

## 4. Discussion

### 4.1. Olive Varietal Composition in the Garda Trentino Area

The area of the Garda Lake within the Trentino province (north of Italy) is the northernmost part of Europe where olive is cultivated. ‘Casaliva’ is claimed as the main variety traditionally grown in the GT from which a world renowned niche EVOO is produced. 

Synonymies of ‘Casaliva’ have been reported among the olive germplasm of other regions of northern (‘Belvedere’, ‘Raza’, ‘Gorgazzo’, ‘Taggiasca’ e ‘Razzola’), central (‘Frantoio’, ‘Correggiolo’, ‘Correggiolo Montegridolfo’ and ‘Correggiolo Pallese’) and southern Italy (‘Ogliarola del Bradano’, ‘Ogliarola Barese’ and ‘Ogliarola Garganica’) [5,6,7,8,21]. ‘Frantoio’ stands out among all these synonymies because it is the most widespread cultivar in Italy. Our results confirm ‘Razza’ and ‘Casaliva’ as ‘Frantoio’ synonymies. Anyway, among the old literature consulted by Hugues [3], there was already a bit of confusion among ‘Razza’ and ‘Casaliva’. ‘Razzar’ or Tuscan ‘Grossajo’ were reported as synonymies of ‘Casaliva’, but some authors maintained that ‘Razza’ corresponded to a cultivar from Umbria with the name ‘Raggia’, instead to the Tuscan ‘Grossajo’. 

Regarding the morphological differences appreciated between ‘Razza’ and ‘Casaliva’, the smaller size of ‘Razza’ olives compared to those of ‘Casaliva’ and a larger canopy (not being the best for the GT edaphoclimatic conditions) were differences already described at the end of the 19th century, features why it was then recommended to graft it with other less demanding varieties such as ‘Casaliva’ [3]. These observations highlight the need of combining molecular identification methods together with a morphological evaluation for the propagation of selected local material and preparation of a nursery production system that conserves the phenotypic intra-varietal diversity that cannot be detected through SSR markers.

‘Casaliva’ has been previously found to be a synonymy of ‘Frantoio’ based on different sets of SSR markers [5,6,7,8]. The reference ‘Casaliva’ accession analyzed here, coming from Lombardy [6], was identical to the reference ‘Frantoio’ except for the UDO43 major allele, as previously observed by [5]. Belaj et al. [62] suggested that multiple bands for UDO43 may be the amplification products of two different loci. In this sense, we only found one genotype with three alleles at this locus (Table S4) and it could also be due to mosaicism, a phenomenon more frequent during the senescent phase of a tree, which can accumulate mutations without phenotypic consequences [20,63,64,65].

Regarding ‘Frantoio’, different molecular variants of this cultivar with the same phenotype compared with the true-to-type genotype, were already previously reported [5,31]. Despite the fact that we have not conducted a systematic and rigorous morphological description of the investigated trees and that the possibility of genotyping errors can never be completely discarded, the genotypes that vary only for one or two alleles from that of ‘Frantoio’ were considered as molecular variants of this cultivar (‘Frantoio’/‘Casaliva’ group, Figure 2). These small genotypic differences could be due to somatic mutations and considered an intra-varietal variation. In fact, until its unique phenotypic and agronomic features are clearly distinguished, a different genotype will not be considered to be a new cultivar (International Union for the Protection of New Varieties of Plants, 1991 in [20]). The number of somatic mutations appearing in a given variety is expected to increase proportionally with its age (the investigated trees were centuries-old) and cultivation area and some genotypes could be more prone to generate somatic variants [65]. Molecular variants have been commonly reported also in other olive cultivars widely cultivated in different areas, as well as in ancient and antique cultivars grown throughout history (i.e., ‘Ogliarola di Lecce’, ‘Ogliarola Barese’, ‘Ogliarola Garganica’, ‘Biancolilla’, ‘Giarraffa’, ‘Moresca’ (Italy), ‘Picholine Marocaine’ (Morocco), ‘Cirujal’, ‘Farga’, ‘Lechin de Granada’, ‘Verdial de Velez Malaga’ (Spain); [17,20,21,31,32,34]. El Bakkali et al. [32] suggested that slight allelic variations are common in cultivars subjected to massive clonal propagation on a spatial or temporal scale, or both.

Therefore, in view of the above, it can be concluded that ‘Casaliva’ from the GT is identical to ‘Frantoio’, being the ‘Casaliva’ from Lombardy variant scarcely represented (our results evidenced that only 1.9% of the analyzed trees presented the reference ‘Casaliva’ profile, while 61% were identical to ‘Frantoio‘, Figure 3). Moreover, considering as intra-varietal variation all SSR profiles within the ‘Frantoio’/‘Casaliva’ cluster (Figure 2), this cultivar results in the predominant genotype in the Garda Trentino olive cultivation area. On the other hand, the fact that all the investigated centuries-old ‘Casaliva’ trees presented the genetic profile of ‘Frantoio’ (81.6%) or ‘Frantoio’ variants (18.4%) evidences the historical bond of this cultivar to the territory. In addition, other cultivars with Tuscan origin were also identified among the ancient olive populations (‘Leccino’, ‘Razzaio’ and ‘Rosselino’) as well as variants with over 90% similarity with Tuscan cultivars, such as ‘Leccino’, ‘Maurino’ and ‘Morchiaio’ [66], which supports the hypothesis that most of the olive cultivars in the GT area were introduced from this region [3,8]. The ‘Razzaio’ genotype (SSR code 91 and 92, Table S4) presented over 95% similarity (Figure 2) with a local accession named ‘Trep’. A cultivar named ‘Trepp’ is reported among the germplasm in the Veneto region [8], although, due to the lack of a genetic profile for comparison, whether it is the same variety or not remains undetermined. ‘Favarol’ is another cultivar once grown in the GT area [3], but the two trees collected under this name clustered distantly from the ‘Favarol’ group of accessions from local collections (Figure 2). The fact that most of the GT genotypes could not be assigned to known cultivars is in agreement with previous reports on ancient genotypes, which suggested that traditional olives are confined in their putative domestication areas [67,68]. Some of them could be feral. This result indicates that the old long-lived trees are reservoirs of genetic diversity preserved by traditional agricultural systems, as observed previously by [20] in southern Spain.

### 4.2. Age Estimate of the Ancient Trees

The strong relationship between trunk size and age of an olive-tree has allowed figuring out different algorithms (developed considering certain environmental and climatic parameters, which determine the annual growth rate, so they have to be optimized for estimation in other cultivation areas) to estimate the age of a tree according to the PBH ([44,45] in [17]). Despite that the algorithms used here were developed for olive-tree age estimation in north-eastern Spain [45] and in Umbria (Italy) [44], they have also been used anyway by other authors to get a rough estimate of the age of the trees [17]. The trunk diameter range (0.57 to 2.0 m) is in agreement with that reported for centennial trees surveyed in south Spain (0.62 to 2.72 m measured at 1 m from the ground) [20] and some of the trees, such as AGT010 Olif de Bòtes and AGT012 Razza del Lenzimot (Table S1), can even be considered ancient monumental olive trees, since they exceeded the 3.5 m of trunk circumference, a threshold established in previous studies [17].

### 4.3. Cultural Practices

The grafting of cultivated olive varieties onto local oleaster or ancient cultivars is a widespread practice [20,69]. Hugues [3] reported that almost all farmers over-grafted ‘Razza’ cultivar because they realized that it did not offer a better yield of oil compared to other cultivars, as it was believed then. ‘Razza’ seemed to be the predominant cultivar in the GT area in the 18th century, since very ancient ‘Razza’ trees that were not possible to graft were reported and the most ancient trees found at that time in the area, such as ‘Gort’, were known as ‘Razza’. Our results showed evidences of this cultural practice. It is likely that scion/rootstock genotype combinations are not randomly distributed, but selected by the growers to introduce genotypes with improved agronomic features and/or better adapted to the edaphoclimatic conditions, as suggested by [70]. The slightly genotypic differences found in some cases between the canopy and the basal part of the tree could be due to a real graft among clones of the same cultivar or to somatic point mutations [20,65].

### 4.4. Population Structure

The joint database of the WOGB germplasm collections of Córdoba and Morocco [32], previously harmonized according to the 13 true-to-type accessions analyzed in common in our laboratory, was used together with the unique genetic profiles obtained from the GT area to better understand the population structure of these genotypes in a context of worldwide diversification of the *Olea europaea* subsp. *europaea*. Despite data harmonization of allele sizes between databases produced comparable results, some discrepancies can be observed anyway [29,30,71]. However, the approach of combining data from available published databases with analyzed own data by harmonizing the external database through the analysis of some reference material has already been previously adopted when population structure of local, regional or small analyzed datasets want to be examined in a wider context [8,72]. 

Comprehensive studies about the Olive germplasm in the Mediterranean Basin based on SSR data revealed that it is structured into three main ancestral cultivated genepools corresponding to three geographic areas: West, Central and East Mediterranean Basin (Q1, Q2 and Q3, respectively; [31,32,73,74]. The Eastern pool (Q3) harbors the eastern maternal lineage (E1), while the other two pools (Western and Central Mediterranean Basin) presented both western and eastern maternal lineages (E1, E2, and E3; [74,75]). Whether admixture between the two wild ancestral olive pools (western and eastern oleasters) occurred before domestication or whether early domestication of the western wild oleaster took place followed by introgression from Q3 cultivars (Eastern Mediterranean) is still not clear [73,74,75]. However, a recent population analysis, based on a genome re-sequencing approach, supports the hypothesis of two independent events in olive domestication, including an early possible genetic bottleneck, and provides evidence that the clustering into different groups (a similar clustering of cultivated accessions into Q1, Q2 and Q3 groups was also observed with this approach) may indicate, not only a strong geographical component, but at the same time a possible phenotypic selection for traits such as fruit size [76]. Our population subdivision at K = 3 was congruent, as expected, with these three genepools and evidenced a high genetic diversity within cultivars putative from the Central Mediterranean Basin (Q2), especially from France, indicating a high admixture level as previously reported by [77]. Despite the high admixture level within the putative Italian germplasm, this was not observed among the GT genotypes, for which 77% of them clustered in the Q2 subpopulation (Figure 4, Tables S7 and S8). This might reflect the antique presence of olive cultivation in the GT area, since cultivars with a historical cultivation tradition, such as ‘Frantoio’, and probably also others not identified but likely used locally (suggested by the old age of the examined trees as well as by the grafting events, see above) can have been selected as the best adapted to the agro-climatic conditions of the GT and successively propagated as elite cultivars in this area [20,73].

### 4.5. Deteriorated Embryos

The rate of deteriorated embryos in ‘Casaliva’ trees in this study was generally slightly higher than the values already reported for the cv. ‘Frantoio’ by [78], while the mean rate of degenerated embryos (35.3%) is in line with the modest germination rates of ‘Casaliva’/‘Frantoio’ seeds previously reported in the literature (between 40% and 50%, [79]). Anyway, this rate was higher in mature (34.9–49.1%) rather than in young trees (28.4%), which might indicate that this phenomenon is more frequent in olive-trees in a senescent phase. Higher rates of deteriorated embryos were observed anyway for those not subjected to any soil management system. According to these observations, the high percentage of degenerated embryos could possibly be related to any effect of plant stress due to water scarcity and nutritional deficiencies, altering the olive vegetative-productive balance and preventing the start of cell division of the embryo, thus causing its degeneration [69]. In fact, zinc deficiency has been associated with programmed cell death (PCD)-related embryo lethality in Norway spruce [80]. Anyhow, the presence of recessive lethal alleles segregating at embryo viability loci, like in the conifer ‘embryo lethal system’ (ELS) model [81], cannot be excluded.

### 4.6. Parentage Analysis and Self-(in)Compatibility of ‘Casaliva’

The number of markers used for the parentage analysis was considered sufficient because, according to the panel constructed by [82], 12 markers were needed to obtain an exclusion probability exceeding 99% when only the offspring and putative parents were tested, however only six markers were needed to obtain the same probability when a known parent was also included, just like in this case. 

Parentage analysis proved to be a useful supplementary tool to drive the choice of pollinizers and to complement, eventually, the study of the local biodiversity of olive. In fact, it allowed the identification of some effective pollen donors, a useful piece of information in order to increase production and avoid the choice of inter-incompatible pollinizers, and, indirectly, it also revealed, through the percentage of unassigned parents, the presence on the territory or in neighboring regions (Venetian and Lombard areas of the Garda Lake) of additional varieties. 

The prevalence of some paternal alleles in embryos deriving from a single plant, suggests that the proximity of effective pollinizers has a fundamental role in the competition between pollen grains [83,84]. 

Regarding the low rate of self-pollination of ‘Casaliva’, the cultivation of which amounted to almost monoculture in the GT, some assumptions can be made. Conflicting results are reported in the literature about self-incompatibility in ‘Casaliva’ (and its synonymies ‘Frantoio’, ‘Ogliarola Barese’) ranging from totally self-incompatible [85,86], to mostly self-incompatible [87], to partially self-compatible [88,89,90] to self-compatible [91,92]. This observed variability in different areas of cultivation of ‘Frantoio’ may suggest an effect of the climate on self-pollination capability. 

In most well-studied cases, self-incompatibility (SI) is controlled by a single locus S with, generally, multiple alleles (or, less frequently, by two independent multiallelic loci [93]). This locus contains at least two genes: a female and a male determinant (expressed in the pistil and the pollen, respectively). The recognition of self or non-self pollen occurs through the interaction between the protein products of these two genes, with the incompatibility response that is triggered when the two determinants come from the same S haplotype [93]. Self-incompatibility mechanisms can be gametophytic (GSI) or sporophytic (SSI). The GSI system is the most widespread in nature [94] and has also been suggested in olive [95,96]; nevertheless, this model does not fit to the different cases of partial or total self-fertility described in this species. Recent studies [90,97,98,99] instead have identified the SSI model as the most adherent to olive SI, but, despite that several S-allele pairs have been identified [98,99], the molecular mechanisms behind SSI are still unknown. Eventually a homomorphic, diallelic self-incompatibility (DSI) system has been described [100,101]. 

An explanation of the low self-fertilization levels found in the cultivar ‘Casaliva’, which is essentially in monoculture conditions in our survey, could be due to a cryptic self-incompatibility (CSI)-like behavior. According to the cryptic self-incompatibility (CSI) model, the reduction in the success of self pollen is due to a lower rate of pollen germination and pollen tube growth compared to outcross pollen (that outcompetes self pollen) [102]. This strategy is a means of preventing inbreeding in the presence of outcrossed pollen and providing reproductive assurance in the absence of pollinizer trees. In fact, experiments carried out on self-fertile cultivars report higher rates of fruit setting obtained through cross-pollination [37,38,39,40,103], being, therefore, the choice of pollinizers also important in self-fertile varieties. As a proof of this, the percentage of self-pollination in the isolated olive trees in Torbole was averagely five-fold higher than those recorded in the commercial olive groves in the other four localities. Accordingly, Quero et al. [103] reported a slower tube elongation under self-pollination in olive. This ‘delay’ would prevent ovule self-fertilization by degeneration of the embryo sac before the pollen tube reaches it and may explain the high number of deteriorated embryos recorded in our study. Furthermore, in olive, self-pollination might be somehow disadvantaged by the fact that ovary receptivity begins even before the opening of the anthers, lasting for five to seven days, whilst the maximum emission of pollen occurs three or four days after the opening of the flowers [104]. Nevertheless, an asynchronous opening of the flowers among the different plants of the same variety and even on different branches of the same plant has been reported [38,105], so that this effect would be maximized in the ‘classical’ trials with bagged branches for the assessment of self-pollination. Lastly, high temperatures during flowering (30–35 °C) have been described to induce inhibitory effects on the germination of pollen and the growth of the pollen tube [106,107] and to increase the level of self-incompatibility and inter-incompatibility in some combinations of cross-pollination [89,108].

Therefore, despite that self-pollen compatibility is genetically controlled and genotype-dependent, other factors such as the synchronism of the respective phases of flowering, the presence of inherent mechanisms that favor cross- against self-pollination and the climatic conditions of cultivation might enhance or attenuate the intrinsic self- and/or cross-pollination capacity of a cultivar influencing its fitness (reproductive success) as a pollinizer; the reason why it is important to evaluate both self- and cross-(in)compatibility upon the environmental and climatic conditions of each cultivation area. In this respect, it seems that ‘Casaliva’ pollen in the GT is self-compatible, presenting this cultivar, thus, a potential self-pollination capability that becomes enhanced or attenuated depending on the factors/conditions described above; although cross-pollination, when possible, appears to be the preferential system.

Regarding the occurrence of a polyembryonic seed (Figure S4), a phenomenon called monozygotic cleavage polyembryony (MCP), it has been previously described in olive by [109].

## 5. Conclusions

The exhaustive genetic study of the old olive trees present in the GT area has evidenced the existence of genotypes not yet cataloged in the wide databases consulted. These hidden olive genetic resources are reservoirs of genetic diversity preserved in an isolated traditional olive growing area. The low level of admixture of the ancient trees investigated compared with the Italian germplasm confirms, in fact, the historical and traditional olive growing culture in this northern area of Italy. Anyway, ‘Casaliva’ is the predominant cultivar in the zone and the analysis of ‘Casaliva’ embryos has revealed a preferential cross-pollination system over self-pollination in this cultivar, despite that it has self-fertility potential. Parentage analysis resulted in a valid supporting tool to drive the choice of effective pollinizers and provided evidence of functional local biodiversity.

## Figures and Tables

**Table 1:** Parameters of genetic diversity for local collections and ancient populations in our study and for other national and international databases published (worldwide olive germplasm collections of Marracketch and Córdoba - WOGBM and WOGBC, respectively- Italian databases from the CREA-OFA and from Perugia University). N: sample size as number of trees analyzed (number of accessions if different to the number of trees), n: mean sample size per locus, nSSRs: number of nuclear microsatellite loci analyzed, SSR profiles: unique genetic profiles, NA: number of different alleles, MNA: mean number of alleles per locus, Ho: observed heterozygosity, He: expected heterozygosity, PA: number of private alleles, PI: Probability of Identity, PIC: Polymorphic Information Content, n.a.: not available.

Population/Database	N	nSSRs	Genotypes Represented by Only One Tree	Genotypes Represented by More Than One Tree	SSR Profiles	NA	MNA	Ho	He	PA *	PI	PIC
Local collections (set of 21 SSRs)	108	21	49	20	69	180	8.57	0.6202	0.6959	29	2.3 × 10^−20^	0.655
Local collections (set of 20 SSRs)	108	20	n.a.	n.a.		169	8.45	0.6090	0.6900	n.a	3.4 × 10^−19^	0.649
Ancient populations	106 (205)	20	35	9	44	131	6.55	0.6877	0.6383	33	4.4 × 10^−16^	0.581
WOGBM (Haouane et al. 2011)	561	12	n.a.	n.a.	505	210	17.5	0.76	n.a.	24	2.6 × 10^−14^	0.737
WOGBC (Trujillo et al. 2014)	824 (499)	33	n.a.	n.a.	332	466	14.12	0.65	0.69	67	n.a	0.650
Italian CREA-OFA (Muzzalupo et al. 2014)	489	11	n.a.	n.a.	439	84	7.6	0.605	0.664	3	n.a	n.a
Italian Perugia (Mousavi et al. 2017)	370	10	n.a.	n.a.	59	126	12.6	0.808	0.802	n.a.	n.a	0.781
Venetian olive germplasm (Hmmam et al. 2018)	239	10	n.a.	n.a.	57	108	11.1	0.914	0.743	n.a.	n.a	0.749
WOGB (El Bakkali et al. 2019)	1091	20	n.a.	n.a.	672	407	20.35	0.746	0.758	43	n.a	0.725

* Measured as alleles observed once for WOGBM, WOGB, local collections and ancient populations, as present only in a single genotype for the WOGBC and as alleles with a frequency <1% for the Italian CREA-OFA database.

## Supplementary Materials

The following are available online at https://www.mdpi.com/2073-4425/11/10/1171/s1, Figure S1: Percentage of the 150 trees from modern orchards that presented each category of genotypes: ‘Frantoio’, molecular variants of ‘Frantoio’/‘Casaliva’, other identified genotypes in the databases consulted and other genotypes remained unidentified. Figure S2: Percentage of deteriorated embryos in modern and mature olive groves and for olive trees not intended for commercial purposes. Different capital letters indicate a statistical difference at *p* < 0.01 (Tukey multiple range test). Figure S3: Percentage of embryos from ‘Casaliva’ auto-pollination in each sampling area. Different lowercase letters indicate a statistical difference at *p* < 0.05 level (Tukey multiple range test). Figure S4: Diembrionyc seed originated from cross pollination of “Casaliva” × “Cipressino”. Table S1: Samples collected from ancient populations in the Garda Trentino area. Table S2: Accessions collected (*n* = 108) for varietal identification purposes from the six local germplasm collections inspected grouped by their genotype for 21 microsatellites markers analyzed. Table S3: Genetic profiles of the reference material analyzed with the set of 21 SSR markers. Table S4: Unique genotypes identified among the local germplasm collections and the ancient populations set of samples for the set of 21 SSR markers analyzed. Table S5. Genetic diversity statistics for each locus. N: Sample Size, Na: No. Alleles, Ne: No. Effective Alleles, Ho: Observed Heterozygosity, He: Expected Heterozygosity, PIC: Polymorphic Information Content, PI: Probability of Identity and r: null alleles’ estimation. n.a.: not analyzed. Table S6: Profiles found for seven microsatellite markers analyzed in the 151 samples collected from modern orchards. Table S7: Assignment of each sample to a cluster (subpopulation) according to the coefficient of membership threshold (Q > 80%) for each K value inspected. Table S8: Subpopulation assignment for samples within its putative geographic origin.
