# Supplementary figures and images for: Genetic Resources of Olea europaea L. in the Garda Trentino Olive Groves Revealed by Ancient Trees Genotyping and Parentage Analysis of Drupe Embryos
Source: Genes (Basel). 2020 Oct 6;11(10):1171. doi: 10.3390/genes11101171 (PMC7600466; doi:10.3390/genes11101171)

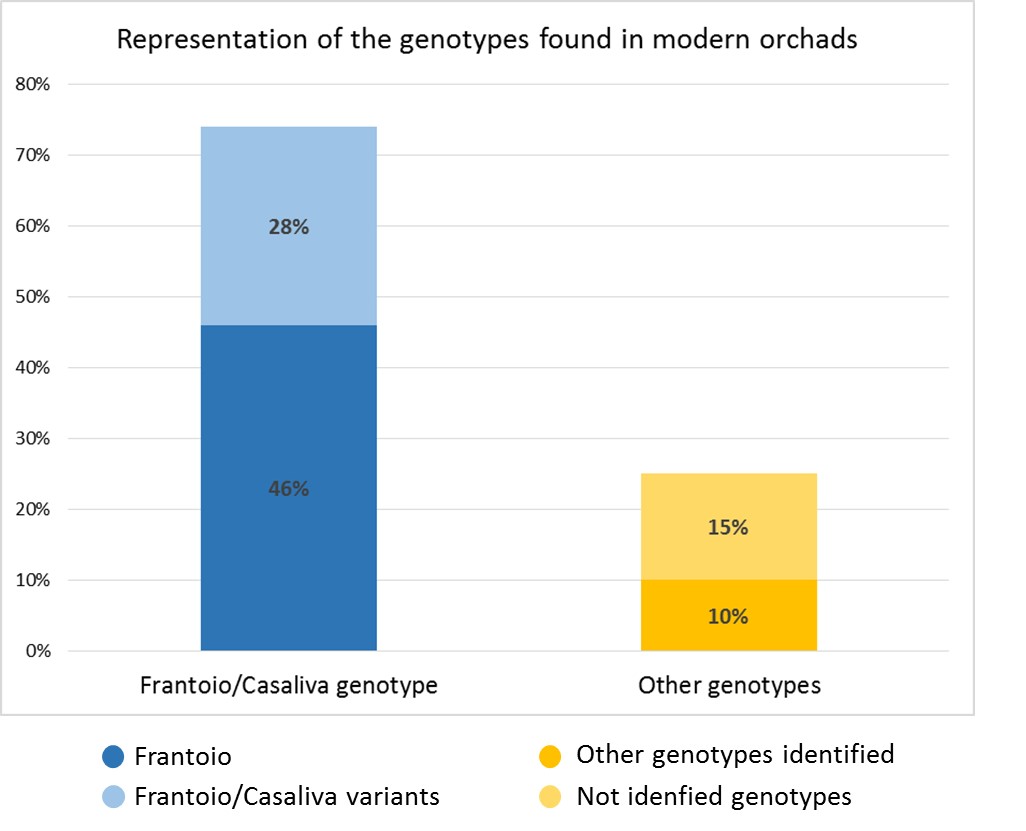

Supplement: Supplementary file 1 [file genes-11-01171-s001.zip › new Figure S1.jpg]

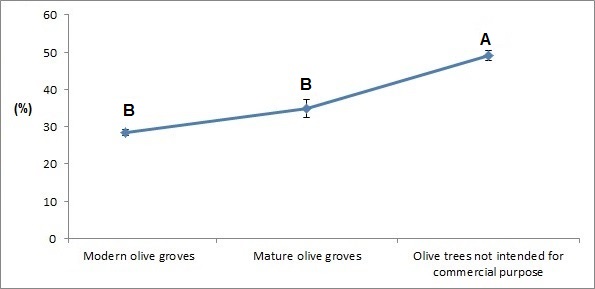

Supplement: Supplementary file 1 [file genes-11-01171-s001.zip › new Figure S2.jpg]

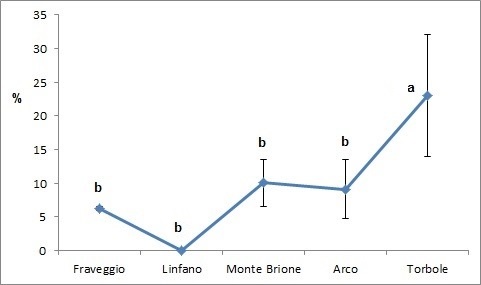

Supplement: Supplementary file 1 [file genes-11-01171-s001.zip › new Figure S3.jpg]

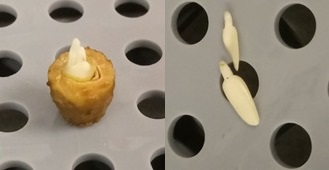

Supplement: Supplementary file 1 [file genes-11-01171-s001.zip › new Figure S4.jpg]
